# Technical advances in global DNA methylation analysis in human cancers

**DOI:** 10.1186/s13036-017-0052-9

**Published:** 2017-03-01

**Authors:** Basudev Chowdhury, Il-Hoon Cho, Joseph Irudayaraj

**Affiliations:** 1 Department of Medicinal Chemistry and Molecular Pharmacology, Purdue University, West Lafayette, 47907 IN USA; 2 Department of Biomedical Laboratory Science, College of Health Science, Eulji University, Seongnam, 461-713 Republic of Korea; 3 Bindley Bioscience Center, Purdue University, West Lafayette, IN 47907 USA

**Keywords:** 5-methylcytosine (5mC), 5-hydroxymethylcytosine (5hmC), 5-formylcytosine (5fC), 5-carboxylcytosine (5caC), LC-MS/MS, Immunoquantitation, Next generation toolset

## Abstract

Prototypical abnormalities of genome-wide DNA methylation constitute the most widely investigated epigenetic mechanism in human cancers. Errors in the cellular machinery to faithfully replicate the global 5-methylcytosine (5mC) patterns, commonly observed during tumorigenesis, give rise to misregulated biological pathways beneficial to the rapidly propagating tumor mass but deleterious to the healthy tissues of the affected individual. A growing body of evidence suggests that the global DNA methylation levels could serve as utilitarian biomarkers in certain cancer types. Important breakthroughs in the recent years have uncovered further oxidized derivatives of 5mC - 5-hydroxymethylcytosine (5hmC), 5-formylcytosine (5fC) and 5-carboxylcytosine (5caC), thereby expanding our understanding of the DNA methylation dynamics. While the biological roles of these epigenetic derivatives are being extensively characterized, this review presents a perspective on the opportunity of innovation in the global methylation analysis platforms. While multiple methods for global analysis of 5mC in clinical samples exist and have been reviewed elsewhere, two of the established methods - Liquid Chromatography coupled with mass spectrometry (LC-MS/MS) and Immunoquantification have successfully evolved to include the quantitation of 5hmC, 5fC and 5caC. Although the analytical performance of LC-MS/MS is superior, the simplicity afforded by the experimental procedure of immunoquantitation ensures it’s near ubiquity in clinical applications. Recent developments in spectroscopy, nanotechnology and sequencing also provide immense promise for future evaluations and are discussed briefly. Finally, we provide a perspective on the current scenario of global DNA methylation analysis tools and present suggestions to develop the next generation toolset.

## Background: The trail of DNA methylation derivatives

In 1866, Gregor Mendel published his seminal research detailing the laws of inheritance [1] and shortly afterwards in 1869 Friedrich Miescher discovered the enigmatic compound “nuclein” or DNA as we know it today [2]. In the first half of the 20th century, Conrad Waddington designated the term “epigenetics” to describe “the branch of biology which studies the causal interactions between genes and their products, which bring the phenotype into being” [3] and used the “epigenetic landscape” metaphor to describe events contributing to embryonic development [4]. The “Sequence Hypothesis” proposed by Francis Crick in 1958 [5] was ultimately established as the “Genetic Code” by research efforts of Marshall Nirenberg, Har Gobind Khorana and Robert Holley [6]. While the genetic code lays out the procedure for translating hereditary information stored in DNA into functional attributes, the natural laws pertaining to “regulation of gene expression” or commonly referred to as the “epigenetic code” are still not understood. The explorative successes of post-1960 research have no doubt enhanced the current knowledge about the diversity of epigenetic mechanisms and its relevance in cancers [for a comprehensive understanding of the history of epigenetics refer to [7]], but as suggested by Bryan Turner much more needs to be done in terms of characterization of epigenetic marks and delineating their biological functions, before the epigenetic code can be deciphered [8].

**Fig. 1:**
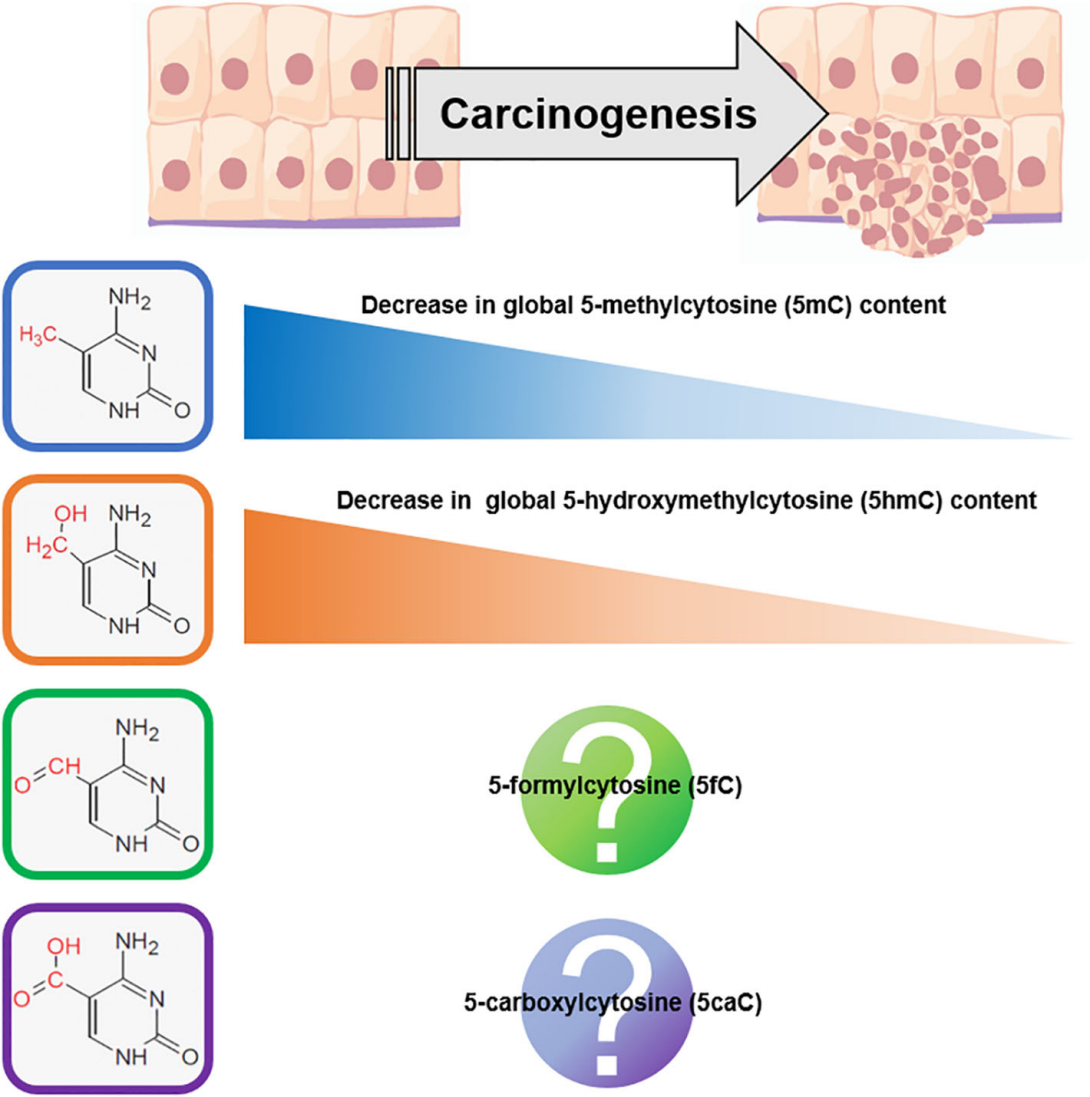
Summary of the status of global levels of the DNA methylation derivatives in normal and tumorous tissue. (Refer to Table 1 for details and references)

DNA methylation is the most widely characterized epigenetic mechanism involved in the regulation of gene expression. Biochemically, DNA methylation refers to the enzymatically (DNA methyltransferases; DNMT 1/3A/3B/3 L) catalyzed addition of a methyl (−CH_3_) group to the C5 position of the cytosine base in DNA resulting in generation of 5-methylcytosine (5mC) (Fig. 1). Conserved across the evolutionary hierarchy, 5mC regulates gene activity in a heritable manner without altering the primary DNA sequence and has been implicated in numerous biological processes [for a comprehensive review refer to [9]]. In healthy individuals, the traditional epigenetics paradigm was based on the association of elevated methylation (hypermethylation) with transcriptionally silent oncogenes and DNA repeat elements. In 2009, Tahiliani et al. discovered that 5mC can undergo Ten-eleven Translocation (TET) enzyme mediated oxidization to 5-hydroxymethylcytosine (5hmC) [10]. The emergence of 5hmC as an epigenetic player disrupted the simplicity of the traditional epigenetics paradigm and called for re-evaluation of the methylation landscape particularly because the tools hitherto used to assay 5mC were not specific and could not discriminate between the effects conferred by 5mC and 5hmC [11]. In 2011 Ito et al. demonstrated that TET can catalyze 5hmC to further oxidized derivatives- 5-formylcytosine (5fC) and subsequently 5-carboxylcytosine (5caC) [12] While the biological significance of these oxidized derivatives of 5mC is still in the process of being uncovered, it is becoming increasingly evident that the dynamic DNA methylation derivatives coordinate among themselves and with other players to regulate gene expression [9, 13–17].

## Relevance of the expedition: Global “hypomethylation” in cancers is almost universal

The global loss of DNA methylation content in human tumors compared to normal tissues was reported in 1983 through independent research efforts of Feinberg et al. and Gamasosa et al. [18, 19]. This novel discovery was initially disregarded as “an unwelcome complication” [20] but research spanning the last three decades has confirmed that the trend of global hypomethylation in human cancers is almost universal [20–24], although each cancer type may have characteristic localized regions associated with hypermethylation or hypomethylation [25–27]. The association between global hypomethylation in cancers and the overarching loss of genomic integrity suggested by chromosomal abnormalities associated with mutations in DNMTs and misregulated methylation patterns over DNA damage repair genes/retroposon elements [9, 21, 28, 29] indicate that it is likely that these events contribute to maintenance of a catastrophic physiological state symptomatic of cancers.

Alterations to 5hmC have also relatively recently been documented in hematological malignancies [30] and other solid tumors. The reduction in the content of 5hmC in adult glioblastoma and astrocytomas has been observed to correlate with poor prognosis [31, 32]. Carcinomas of the lung [33], prostate [34], breast [34, 35], liver [33, 36], kidney [33], esophagus [37] and colon [34]; adenocarcinomas of the pancreas [33], prostate [33], stomach [33], uterus [33], and ovary [33] showed a significant reduction of 5hmC levels compared to corresponding normal tissues [34]. In comparison to 5mC and 5hmC, little is known to impute the trend of global 5fC and 5caC levels in tumors. While a recent study reported elevation of 5caC levels in invasive ductal carcinoma and glioma [38], another study noted the depletion of 5fC and 5caC in colorectal carcinoma [39]. Table 1 provides a summary of the recent clinical studies in global analysis of DNA methylation derivatives and drives home their relevance in the context of human cancers.

**Table 1:** Summary of representative clinical studies performed during the period (2011–2016) to estimate global levels of DNA methylation derivatives. Abbreviations: FFPE- Archived Formalin-fixed, Paraffin-embedded; IHC- Immunohistochemistry; Liquid chromatography-electrospray ionization-tandem mass spectrometry (LC-ESI-MS)

Epigenetic Mark	Method of Study	Tumor type	Observation	Clinical relevance of observation
5mC	IHC	Colorectal cancer (*n* = 30) Vs Control group (*n* = 30)	Loss of 5mC	Associated with advanced colorectal adenomatous polyps [88]
5hmC	IHC	Clear cell renal cell carcinoma (*n* = 111) Vs matched adjacent tissue	Loss of 5hmC	No correlation with grade/prognosis [89]
5hmC	IHC	Urothelial cell carcinoma (*n* = 55) Vs matched adjacent tissue	Loss of 5hmC	No correlation with grade/prognosis [89]
5mC & 5hmC	LC-ESI-MS	Clear cell renal cell carcinoma (*n* = 36) Vs paired normal	Loss of 5hmCNo change in 5mC	No correlation with grade/prognosis [90]
5mC	IHC	Tongue squamous cell carcinoma (TSCC) (*n* = 248) Vs Tumor adjacent normal (TAN) (*n* = 235)	Loss of 5hmC	Associated with the poor disease-specific survival in TSCC patients [91]
5mC	ELISA	Renal Cell Carcinoma (*n* = 889) Vs age, gender, ethnicity matched control group (*n* = 889)	Loss of 5mC	Associated with risk of developing RCC [92]
5mC	LINE1pyrosequencing	Hepatocellular carcinoma (*n* = 208)	Loss of 5mC	Associated with poor disease free survival [93]
5mC	LINE1 LUMA	Leukocytes of Breast cancer patients (*n* = 384) Vs matched control (*n* = 384)	Loss of 5mC	Associated with occurrence of cancer regardless of hormone receptor status [94]
5mC	LINE-1 Pyrosequencing	Colorectal cancer with liver metastases (*n* = 42) Vs matched primary (*n* = 24)	No change	No correlation [95]
5mC	LC-MS/MS	Laryngeal cancer (*n* = 72) Vs adjacent normal laryngeal tissue (*n* = 72)	Loss of 5mC in both groups	No correlation [96]
5mC	IHC	Prostate Cancer (*n* = 48) Vs adjacent benign (*n* = 48)	Loss of 5mC	No correlation with prognostic /pathologic grade [97]
5hmC	IHC	Parathyroid carcinoma (*n* = 17) Vs Parathyroid adenoma (*n* = 43)	Loss of 5hmC	Diagnostic criterion for rare disease [98]
5hmC	LC-MS/MS	Bone marrow & Blood from AML (*n* = 206) Vs Healthy control	Wide range of 5hmC	High 5hmC levels associated with poor prognosis, low levels have no correlation [99]
5hmC	IHC	Glioblastoma (*n* = 162) Vs healthy control (*n* = 66)	Loss of 5hmC	Marker for tumor infiltration zones [100]
5caC	IHC	Breast cancer (*n* = 59) Vs healthy control (*n* = 28)	Gain of 5caC	No correlation arrived at [38]
5mC, 5hmC, 5fC & 5caC	LC-ESI-MS	Colorectal carcinoma (*n* = 24) Vs matched tumor-adjacent normal	Loss of 5hmC, 5fC and 5caC. No change in 5mC	No correlation arrived at [39]

## Undertaking the expedition: Tools for quantifying DNA methylation derivatives

**Fig. 2:**
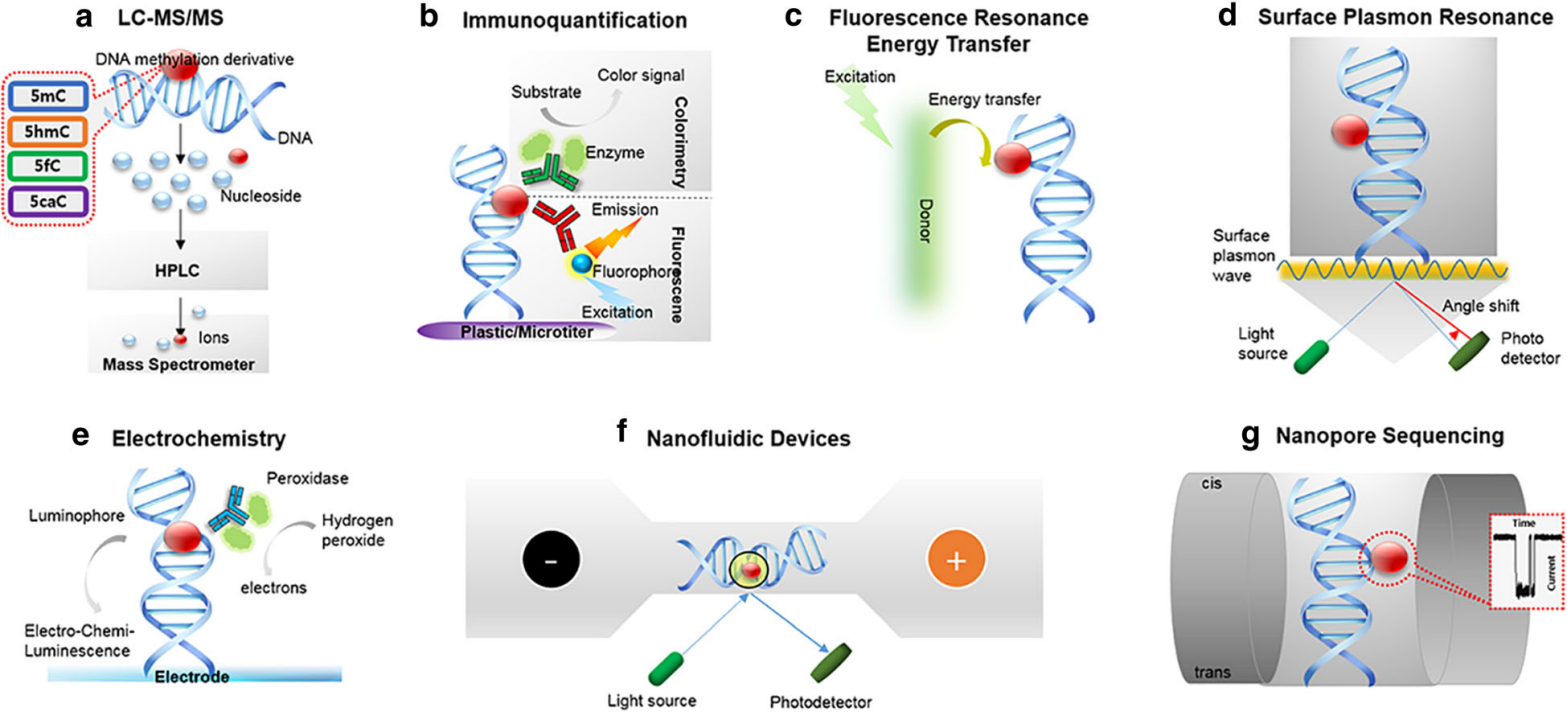
Schematic representation of methods for global analysis of DNA methylation derivatives based on (**a**) LC-MS/MS, (**b**) Immunoquantification, (**c**) FRET, (**d**) SPR, (**e**) Electrochemistry, (**f**) Nanofluidics and (**g**) Nanopore Sequencing

**Fig. 3:**
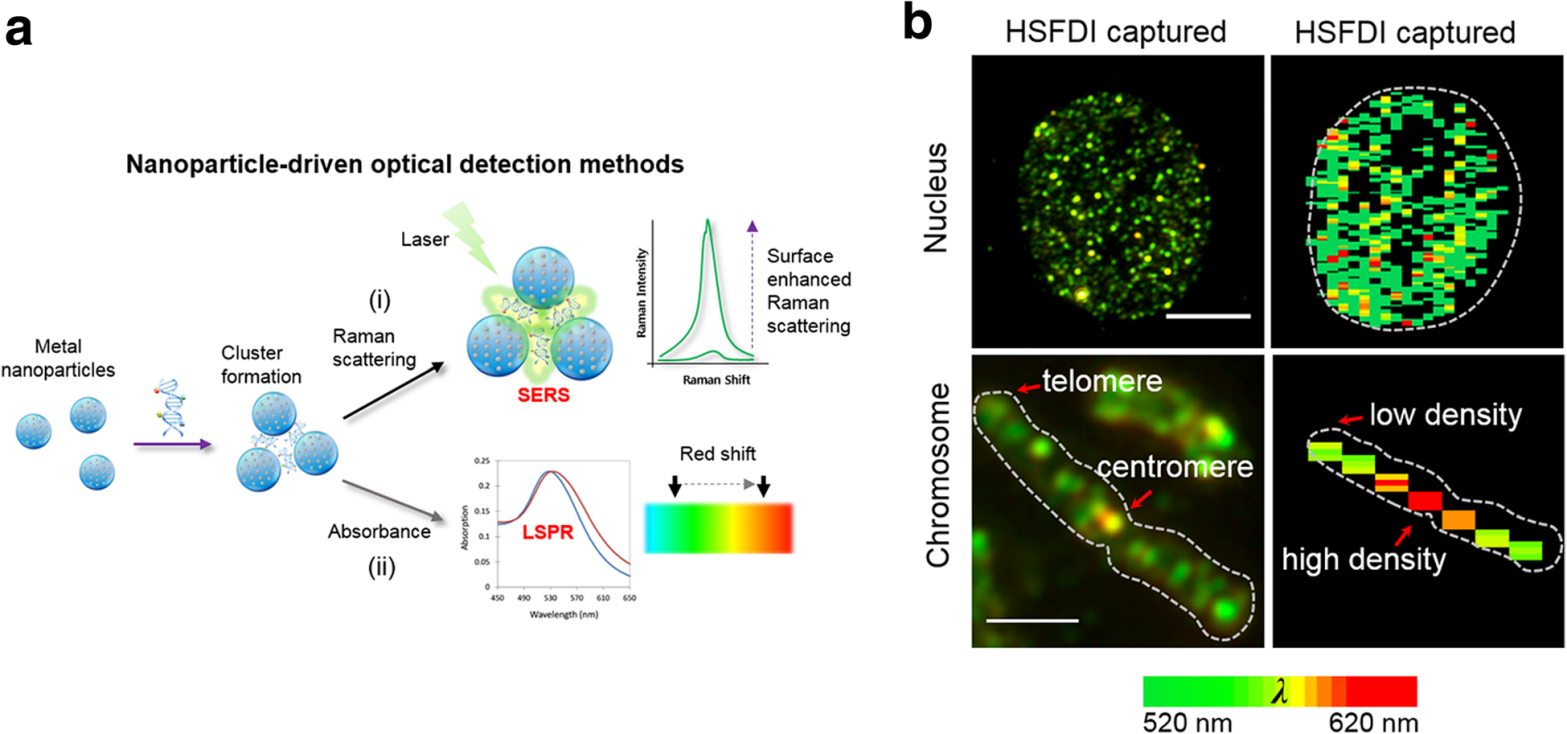
Nanoparticle-driven optical tools for the detection of DNA methylation derivatives (**a**) (i) Surface enhanced Raman scattering (SERS) and (ii) Localized surface plasmon resonance (LSPR). SERS usually occurs on plasmonic nanostructures and dramatically enhances Raman scattering of adsorbed molecules. SERS efficiency is directly related to not only proximal distance among the particles shown here but also size, shape etc. LSPR that describes maximal optical absorption at the plasmon resonant frequency of nanoparticles can be distinguishably changed in the form of cluster formation of nanoparticles. **b** An illustration of quantification of subcellular 5caC (in the context of intact nucleus and single chromosome) with the help of local surface plasmon resonance (LSPR) properties of nanoprobes (nanoparticles conjugated antibody). The figs. on the left represent Hyperspectral dark-field imaging (HSDFI) of 5caC distribution, while the corresponding figs. on the right demonstrate reconstructed spectral maps of 5caC (scale bar = 5 μm) Reprinted with permission from [65] Copyright (2015) American Chemical Society

In 1948, Rollin Hotchkiss while attempting to quantitatively study the composition of the eukaryotic DNA using paper chromatography, reported the incidence of a minor constituent (designated as “epicytosine”) with a migration rate slightly greater than that of cytosine and suggested that the uncharacterized “epicytosine” might be 5mC [40]. Ever since then, chromatographic tools have dominated the field of DNA methylation analysis and have subsequently evolved to include gas [41] and liquid [42, 43] chromatography. Liquid chromatography coupled with mass spectrometry (LC-MS/MS) is regarded as “the gold standard” for quantitative analysis of 5mC and currently this procedure has been adapted to incorporate analysis of 5hmC, 5fC and 5caC. The emergence of immunoquantification tools particularly in clinical settings serves as an alternative strategy for analyzing the four DNA methylation derivatives known as of today. Additionally two other methods based on LINE-1 pyrosequencing [44] and methylation-sensitive restriction digestion [45] are well established for the quantifying of global 5mC in clinical samples but are inapplicable to the analysis of the other DNA methylation derivatives and will not be elaborated in this review. In the following sections we will provide our perspective of the two prominent DNA methylation analysis toolsets based on LC-MS/MS and immunoquantification followed by some strategies that singularly or in combination show great promise of being developed as the next-generation toolset (See Figs. 2 and 3)

### LC-MS/MS tools

In 2005, Song et al. reported a liquid chromatography electrospray ionization tandem mass spectrometry (LC-ESI-MS/MS) [46] based method to quantitate 5mC and prescribed its application to archived tumor specimen as well as clinical samples derived from laser capture micro-dissection owing to a sensitive limit of detection (LOD) of 0.2 fmol and requirement of as little as 4 ng input DNA (Fig. 2a). Kok et al. further developed this method further and utilized the principle of LC-ESI-MS/MS to quantitate 5mC [47] and reported a LOD of 2 pg of cytosine and 5-methylcytosine. After the discovery of 5hmC in the human genome, it became imperative to include its quantitation to evaluate the global methylation landscape and Le et al. developed the liquid chromatography electrospray ionization tandem mass spectrometry with multiple reaction monitoring (LC–ESI–MS/MS–MRM) to simultaneous quantitate the global levels of 5mC and 5hmC [48] with a LOD of 0.5 fmol per nucleoside base.

Recently developed methods based on variations in liquid chromatographic techniques have pushed the limit of epigenetic analysis and have subsequently been modified to include quantification of 5fC and 5caC in addition to 5mC and 5hmC. The discoverers of 5fC and 5caC, Ito et al. adapted the mass spectrometric parameters and reported the LOD to be 5 fmol and 10 fmol respectively [12]. Thereafter to account for the low abundance of 5fC and 5caC, several modifications have been introduced to enhance the detection limits of these derivatives by LC-ESI MS/MS. In 2015, Tang et al. developed a labeling technique involving selective derivatization of cytosine moieties using 2-bromo-1-(4-dimethylamino-phenyl)-ethanone prior to LC-ESI-MS/MS for quantifying all the four known DNA methylated derivatives concurrently in archived Formalin-fixed Paraffin-embedded (FFPE) tumor specimen [49]. The LOD of 5mC, 5hmC, 5fC and 5caC were described as 0.10, 0.06, 0.11, and 0.23 fmol respectively, representing a 35–123 fold enhancement in detection sensitivity compared to LC-ESI-MS/MS without chemical derivatization. In addition, Zhang et al. hydrolyzed genomic DNA by formic acid and analyzed 5caC by hydrophilic interaction liquid chromatography-tandem mass spectrometry (HILIC-MS/MS) [50] yielding an LOD of 0.1 ng/mL in the linear range of 40–4000 ng/ml. Yin et al. was able to demonstrate a 1.8 − 14.3 fold enhancement of the LC-ESI-MS/MS detection of 5fC along with 5mC and 5caC by the use of ammonium bicarbonate (NH_4_HCO_3_) as an additive to the mobile phase [51].

Despite the analytical superiority of LC-MS/MS, the key hindrances to its widespread use in quantifying methylated derivatives arise from the intricate procedures involved in analyzing and maintaining the instrument. Smaller clinics are particularly unwilling to adopt the LC-MS/MS technology owing to the exorbitant prices of the initial installation and the requirement of a highly skilled manpower to oversee daily operations. Compared to immunoassay based techniques which are commercially available in the form of kits with detailed working protocols, LC-MS/MS requires significant investment in terms of time and money in standardizing protocols. However, chromatography techniques are still the standard for pharmacokinetics and pharmacodynamics studies and will continue to dominate this field. Given the surge in epigenetics research we expect a significant effort in this field with emphasis on single cell analysis. Improvements in developing an automated workflow with technical support, will help lower service expenses, generate higher sample throughput and can have a considerable contribution in wider acceptance of LC-MS/MS.

### Immunoquantification tools

Microtiter plate based immunoquantification known as enzyme-linked immunosorbent assay (ELISA) is a well-established method and can be effectively applied for the detection of epigenetic modifications of DNA immobilized on plastic, using an antibody highly specific to the target epigenetic marks (Fig. 2b). As early as 2000, Piyathilake et al. reported suitability of immunoquantification of 5mC over other first generation methylation quantification assays in rare clinical specimen [52]. For 5mC, quantitative analysis by ELISA as well as the semi-quantitative immunohistochemical evaluation in clinical biopsies or cells collected by laser capture microdissection (LCM) offers the advantage of cost and speed. In 2012, Kremer et al. generated a rapid and sensitive ELISA based assay to quantitate 5mC (methDNA-ELISA) [53]. This method requires as little as 10 ng of input genomic DNA, demonstrates linearity in the 1–10% genomic range and correlates well with MS approaches of 5mC quantification. Commercially available antibodies targeting 5hmC, 5fC and 5caC were practically nonexistent in the early years following their discovery, however post 2011 with the generation of highly specific antibodies, immunoassays were gradually adapted to include quantification of these novel epigenetic derivatives as well. Li et al. investigated for the first time the abundance of 5hmC in human tissues by ELISA [54]. This method yields an LOD of 0.1 ng with a dynamic range 0.2–10 ng of 5hmC.

For simultaneous analysis of the four epigenetic marks, Chowdhury et al. designed a biotin–avidin mediated enhanced enzyme-based immunoassay (EEIA) and evaluated its performance in genomic DNA isolated from peripheral blood of patients diagnosed with metastatic forms of lung, pancreatic and bladder cancers [55]. Analytical sensitivity was significantly improved by increasing the number of labeling enzymes facilitating color detection on the antibodies, achieving a LOD of 1–2 pg and enabling the detection of the rare epigenetic marks. EEIA was subsequently utilized to evaluate the extent of alteration of the methylated DNA derivatives upon treatment by Decitabine- an FDA approved DNA demethylating drug in myeloid malignancies [13] as well as by the chemically induced hypoxia agent sodium dithionite [56], indicating the versatility of the assay in multiple contexts. Recently by utilizing the potential of locus specific methylation status to confer conformational differences, Kurita et al. introduced a novel immunochemical approach of performing methylation analysis at single CpG loci on a conventional microtiter plate format [57]. Microtiter plate assay is universal and commercialized by biotech companies such as Epigentek Group, Sigma-Aldrich and Zymo Research. However, the analytical sensitivity of the rarer epigenetic derivatives particularly 5fC and 5caC is variable and often these derivatives remain undetectable. Sample processing and the unknown biological context of these derivatives may in some ways contribute to the unpredictability in detection of these rare marks. Key opportunities to advance this technology is in requiring less input DNA to perform the analysis as well as incorporation of a suitable signal enhancement strategy using well-defined conjugates including nanoparticles, enzymes and fluorophores. Given the familiarity of immunoquantification tools, this approach will continue to be extensively used in methylation analysis primarily due to the relative ease of adaptability in a clinical setting.

### Spectroscopic strategies

There have been many interesting reports on fluorescence-based epigenetic analysis owing to its simplicity for signal generation and detection. Wang et al. demonstrated a particle counting assay for rapid and sensitive detection of DNA modifications using benzo[a]pyrenediol epoxide (BPDE)-DNA adducts that were captured by immuno-magnetic particles [58]. By amplifying fluorescence signal with OliGreen™ dyes, the captured BPDE-DNA adducts could be quantified by particle counting, which was proportional to the modification level in genomic DNA. The detection of limit was about 180 fM. In addition, Feng et al. developed a fluorescence resonance energy transfer (FRET) assay using an optically amplifying cationic conjugated polymer (CCP, poly((1,4-phenylene)-2,7-[9,9-bis(6′-*N*,*N*,*N*-trimethyl ammonium)-hexyl fluorene] dibromide)) [59]. The occurrence of FRET between CCP and fluorescein (Fig. 2c) incorporated into DNA was used for read out, however this assay took about 20 h to attain the methylated level of cancer cells. Zhang et al. utilized an identical method for diagnostic and screening of cancer [60]. Single molecule techniques to monitor the dynamics of epigenetic proteins exist [57, 61] but these are not applicable for routine analysis. Precedence for quantification of epigenetic marks in nucleosomes including resolving the stoichiometry of the epimarks using single cell-based FRET approaches also exist [62] and these tools remain to be optimized for DNA methylation analysis. With advances in microscopy, especially in sensitivity (single molecule techniques) and resolution (super-resolution techniques), basic research will continue to enhance our understanding of the dynamics of epigenetic programming.

As one of the highly sensitive spectroscopic techniques, Surface plasmon resonance (SPR) known for its appeal in monitoring biomolecular interactions have also been applied in epigenetics evaluation (Fig. 2d). Nguyen et al. introduced a strategy for ultrasensitive detection of methylation of ctDNA of PIK3CA gene based on localized SPR (LSPR) associated with plasmon coupling mode of gold nanoparticles[63] to observe a shift in the LSPR peak upon the immunogold colloids binding to two methylcytosines, to yield an extremely low LOD of ~50 fM. Kurita et al. reported a sequence-specific immunoassay chip for DNA methylation by microfluidic surface plasmon resonance (SPR) detection [64]. By utilizing an affinity measurement involving the target, (methyl-) cytosine, in a single-base bulge region and an anti-methylcytosine antibody in combination with a biotinylated bulge-inducing DNA probe, this system could obtain the methylation status in 6 attomoles (48 femtograms) of synthesized oligo DNA in 45 mins, which is the fastest DNA methylation assessment hitherto reported. Darkfield microscopy based on SPR have been implemented by Wang et al. to quantify global methylation levels at the single cell level [65], showing promise as a routine screening tool for in situ analysis in the context of tissues.

### Electrochemical tools

Variations of electrochemical tools based on redox reactions have been introduced for detection of DNA methylation. Kurita et al. introduced methylated cytosine in DNA via ELISA with ECL detection in real genomic sample [66] (Fig. 2e). Here, an acetylcholinesterase was employed as enzyme tracer labeled with anti-methyl cytosine, which converted acetylthiocholine to thiocholine, enabling accumulation on gold electrode surface and quantitatively measurement of 5mC in the range from 1 to 100 pmol. By glycosylation modification of 5hmC with glucosyltransferase and sodium periodate, Chen et al. detected 5hmC at sub-nanogram level, which was 20 times more sensitive than the commercial kit based on optical measurement [67]. Carbon-based nanomaterials such as carbon nanotube and graphene were recently employed as alternative electrodes to the conventional metal electrode due to its high electrical conductivity. Wang et al. reported a polypyrrole (PPyox)-directed multiwall carbon nanotubes (MWNTs) film modified glassy carbon electrode (GCE) which was used to electrically oxidize DNA bases for evaluation of DNA methylation level [68]. Due to extraordinary catalytic property of PPyox/MWNTs/GCE, the peak potential of 5mC was distinctive compared with other bases, especially the unmethylated cytosine, upon applying 180 mV, enabling rapid detection of the methylation status in real samples within 45 min. The major advantage of electrochemical method is limit of detection and miniaturization. Additionally, it can be anticipated that micro-electro-mechanical system (MEMS) and nanotechnology will be combined for miniaturization in the future. However, the lower sample volume may cause low signal-to-noise ratio, thus more elaborate manufacturing process is required.

### Microfluidic tools

Microfluidic platform technology has several advantages over conventional analytical methodologies, enabling fast response, cost effectiveness and low consumption of reagents. Recently this method has been applied in the field of epigenetics to efficiently enhance performance of DNA methylation analysis. Cipriany et al. used fluorescently labeled Methylated DNA Binding Domain (MBD) proteins as probes to perform Single-Chromatin analysis at the nanosacle (SCAN) in DNA restricted to microfabricated nanofluidic channels (Fig. 2f) enabling rapid and real-time interrogation of individual molecules of methylated DNA based on their fluorescent signatures [69]. Ronen et al. presented a universal, high-throughput, microfluidic-based fluorometric method for studying DNA methylation [70], employing bacterial HPAII DNA methyltransferase of which enzymatic activity was analyzed by measuring Michaelis-Menten constant. The values were determined to be 5.8 nM and 9.8 nM respectively. These pioneering efforts paved the road to the realization of epigenetic analysis in microfluidic devices, with a possibility of ultimately utilizing these devices in point-of-care testing. However, despite its advantages over conventional methods, limited work exists in microfluidic-based epigenetic analysis. A possible reason could be the complexity of sample preparation, reliability and robustness of the approach.

### Nanopore Sequencing

Nanopore technology offers a promising alternative to conventional DNA sequencing by measuring distinctive electric currents obtained from different bases and has been recently applied for epigenetic studies. Zeng et al. reported a α-hemolysin-based nanopore sensing method (Fig. 2g) for 5mC and 5hmC detection in DNA at the single-molecule level [71]. Here, 5hmC is first selectively modified with iron-linked crosslinker via Click chemistry. Subsequently the passage of the modified bases through nanopores causes unbinding of the host-guest complex generating characteristic current signatures and enables obtaining quantitative information on the 5mC and 5hmC. Recent studies have focused on evolving an electronic signature of methylated DNA bases [72] as well as development of novel nanaomaterials for fabricating nanopores. The electronic signature based monitoring of modified DNA bases through nanopore has excellent appeal in high throughput, especially considering the state-of-art standardization of manufacturing process in materials research.

### Nanoparticle based tools

Nanoparticles that have been employed as tracers in many biosensor applications have also been employed in exploratory epigenetic research owing to its extraordinary physical properties such as photothermal effect, localized surface plasmon resonance which are based on electromagnetic field passing around the nanoparticle surface. The essence of this approach is the induction of particle aggregation to observe a shift in peak for detection by fluorescence or colorimetry. Ge et al. demonstrated a simple colorimetric method to detect DNA methylation [73]. Here, methylated CpG region was captured and enriched by immunomagnetic separation followed by release via heat denaturation. By controlling salt-induced aggregation process associated with unmodified gold nanoparticle, a LOD of 80 fmol was achieved. This method is semi-quantitative by common UV/Vis spectrophotometer, enabling simple and rapid detection of DNA methylation. Interestingly, nanoparticles can be utilized for enhancing efficiency of isolation of genomic DNA and can be subsequently utilized for methylation analysis. Zhou et al. developed a novel one-point extraction technique from whole blood employing bi-functional carboxyl-functionalized magnetic nanoparticle used as solid-phase adsorbent [74]. Here, the extracted chromatin from leukocytes via magnetic separation was concentrated and coated on a microtiter well and analyzed [75] for the detection of the four different cytosine derivatives. Nanoparticles depending upon the material can be used as reporters in a sensor device. Since the size can be tuned in the range from 10 to 200 nm with slight modification of existing protocols, there are many ways to optimize analytical conditions for epigenetic analysis and utilization of nanoparticles in other detection modalities, such as microfluidics, plasmonics and electrochemical sensing, and in spectroscopy. Fig. 3a demonstrates the basic concept of nanoparticle-based aggregation as a signal for SERS and LSPR sensing.

### Surface enhanced Raman scattering (SERS) based tools

Wang et al. developed a novel concept for enzymatic control of plasmonic coupling for DNA demethylation [76]. Here, gold nanoparticle with a Raman reporter and hemimethylated DNA were used as probes. Destabilized nanoparticles were aggregated among others, which generated strongly distinctive SERS signals in response to DNA methylation. Since this method was performed by a homogenous single step analysis, rapid, convenient and a miniaturized analytical method for epigenetic analysis could be developed. (Fig. 3b) Furthermore silver nanoparticles were also used as SERS-based enhancement substrate combined with hybridization chain reaction for the determination of DNA methyltransferase [77]. Morla-Folch et al. demonstrated the feasibility of direct SERS in combination with chemometrics and microfluidics for the relative quantification of the four DNA methylation derivatives in single- and double-stranded DNA [78]. More recently, Ouyang et al. have shown that detection of 5caC and 5hmC along with 5mC is possible with SERS using a novel graphene wrapped plasmonic material [79]. In the future, enhanced approaches based on nanoparticles or enzymes or development of hand-held units will be more common place. Given the recent work and the advent of new materials and standardization of manufacturing processes, one can expect SERS to become a viable option for routine monitoring of epigenetic events.

## Conclusion and the future roadmap

**Fig. 4:**
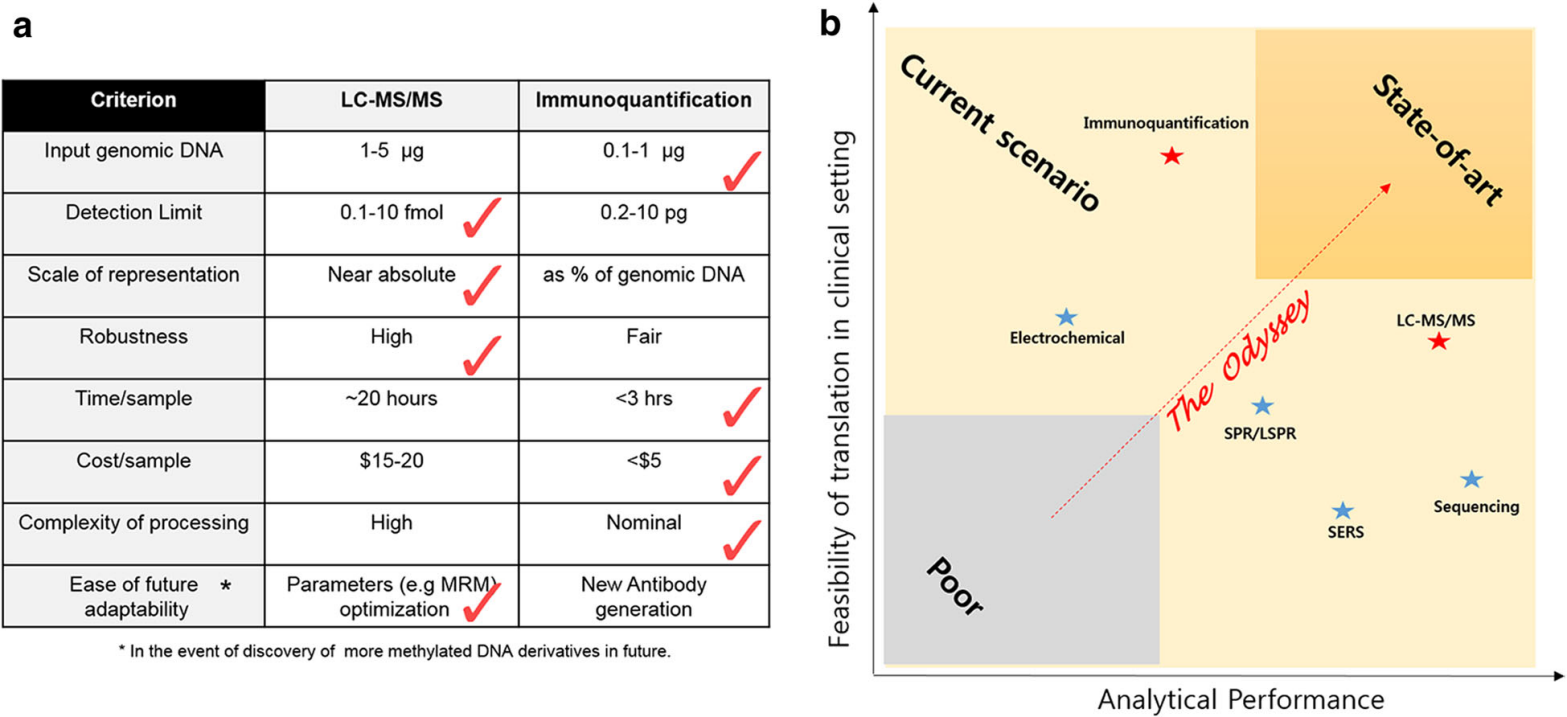
Opportunities for innovating global 5mC analysis methods. **a** Comparison of tools based on LC-MS/MS with immunoquantification to perform global methylation analysis. The red check mark indicates the method that displays superiority on the basis of the indicated criterion. **b** The authors’ illustration of how the current global DNA methylation derivatives’ analysis methods fare on the basis of analytical superiority (aggregate of detection limit, input genomic DNA and robustness wherever available) and feasibility of translation (aggregate of time, complexity and cost)

The challenge of quantifying global levels of DNA methylation derivatives can be gaged from the relative abundance of these epigenetic marks. In humans, 5mC makes up about 1% of the total DNA bases [80] and 5hmC abundance is ∼ 10 to 100-fold lower than that of 5mC [10, 32, 81]. On the other hand, 5fC and 5caC occur ∼ 40 to 1000 times less frequently than 5hmC [12]. For over three decades, chromatography based methods have continued to dominate bioanalytics and it is reasonable to expect that LC-MS/MS will continue to play a critical role in the evaluation of epigenetic modifications. It is worth mentioning that 5hmC, 5fC and 5caC were first discovered in human tissues by thin-layer chromatography and finally confirmed by LC-MS [12, 82]. Immunoquantification tools serve as a simple strategy and remain extensively used for the analysis of global content of methylated DNA derivatives especially in the post-2011 era and serves as an invaluable tool for clinicians. Additionally, tools analyzing the 5mC levels of DNA repetitive elements such as LINE-1, Alu and Sat-α can serve as acceptable surrogate indexes to estimate global DNA methylation level [83, 84] but cannot be applied to the analysis of the other derivatives. Thus, LC-MS/MS and immunoquantification constitute the two most widely exploited methodologies for global analysis of DNA methylation derivatives in human cancers. While, LC-MS/MS is considered as the gold standard method for quantitative analysis of DNA methylation derivatives, the logistical and technological complexities involved in processing and analyzing data, limits its applicability in a clinical setting. On the other hand, immunoquantification is simple and can be successfully integrated with emerging optical, electrochemical and microfluidics technologies, but fares only second to LC-MS/MS in terms of analytical metrics (details of the comparison depicted in Fig. 4a). The sophistication in optics, antibody development methods, advances in materials research, standardization of materials processing methods, scaleup of nanoparticle fabrication processes presents enormous opportunities for further refinement of 5mC analysis. It is conceivable that rapid point of care (POC) epigenetic screening methodologies based on the emerging technologies will be developed in the near future (please refer to Fig. 4b to understand the authors’ illustrative summary of the current state of global DNA methylation analysis tools and the opportunities for development of state-of-art analysis tools).

It is also conceivable that with the emergence of Next Generation Sequencing (NGS) technologies, quantification of global methylation derivatives along with the precise identification of localized sites undergoing these alterations will become prevalent. While this may in the foreseeable future help clinicians make informed choices pertaining to patient profiling and therapeutic management, standards will have to be developed to decorously interpret the disease risk imparted by global changes of the methylome. We realize that the infrastructural wealth available to scientists in biomedical settings may not be practical in a clinic and in this regards, and to address this challenge our lab and others have used lateral flow techniques that can potentially be used for onsite sensing [85, 86] in conjunction with chromatin extraction methods [87] to addressing this lacunae. Sample preparation will continue to challenge the point of care sensors development. However, we are optimistic that advances in miniaturization, development of novel materials, production of capture biomolecules (antibodies, aptamers etc.) will infuse sufficient enthusiasm to further the field of developing analytics for epigenetics. Finally, further explorations of the molecular dynamism of 5hmC, 5fC and 5caC will bring clarity to their biological significance in cancers and identify other areas for the development of tools for diagnostic determination of the methylated DNA derivatives. We expect loci-specific evaluation and quantification of epigenetic targets utilizing modern technologies to become important metrics with more mechanistic studies. In this regard, development of algorithms with heuristics to expound on the profiles of methylome for prognostic determination could become prominent. In summary, it is exciting to note the milestones covered in this trail of DNA methylated derivatives and assess from these studies the impending way ahead for developing tools that hold the key to understanding the “epigenetic code” and its deregulation in diseases such as cancer.
